# Systemic maternal inflammation promotes ASD via IL-6 and IFN-γ

**DOI:** 10.1042/BSR20220713

**Published:** 2022-11-16

**Authors:** Daniel Majerczyk, Elizabeth G. Ayad, Kari L. Brewton, Pichrasmei Saing, Peter C. Hart

**Affiliations:** 1 College of Science, Health and Pharmacy, Roosevelt University, Illinois 60173, U.S.A.; 2 Loyola Medicine, Berwyn, Illinois 60402, U.S.A.

**Keywords:** ASD, autism-spectrum disorders, interferon-gamma, interleukin 6, maternal immune activation, MIA

## Abstract

Autism spectrum disorder (ASD) is a neurological disorder that manifests during early development, impacting individuals through their ways of communicating, social behaviors, and their ability to perform day-to-day activities. There have been different proposed mechanisms on how ASD precipitates within a patient, one of which being the impact cytokines have on fetal development once a mother’s immune system has been activated (referred to as maternal immune activation, MIA). The occurrence of ASD has long been associated with elevated levels of several cytokines, including interleukin-6 (IL-6) and interferon gamma (IFN-γ). These proinflammatory cytokines can achieve high systemic levels in response to immune activating pathogens from various extrinsic sources. Transfer of cytokines such as IL-6 across the placental barrier allows accumulation in the fetus, potentially inducing neuroinflammation and consequently altering neurodevelopmental processes. Individuals who have been later diagnosed with ASD have been observed to have elevated levels of IL-6 and other proinflammatory cytokines during gestation. Moreover, the outcome of MIA has been associated with neurological effects such as impaired social interaction and an increase in repetitive behavior in animal models, supporting a mechanistic link between gestational inflammation and development of ASD-like characteristics. The present review attempts to provide a concise overview of the available preclinical and clinical data that suggest cross-talk between IL-6 and IFN-γ through both extrinsic and intrinsic factors as a central mechanism of MIA that may promote the development of ASD.

## Introduction

Autism spectrum disorder (ASD) is a neurodevelopmental disorder estimated to effect ∼1.5% of individuals in developed countries with an average age of diagnosis ∼3 years [1]. The defining clinical characteristics of ASD include abnormal repetitive behaviors, deficient or absent social interactions, and impaired cognitive flexibility [2]. Patients with ASD also vary in the frequency and severity of comorbid neurobehavioral disorders (e.g., depression and anxiety) and intellectual deficits [1]. While the etiology of ASD is poorly understood, it has often been postulated to result in part from neuroinflammation in different regions of the brain [3]. Autoimmune disorders and immune activation in response to exposure to various antigens, whether environmental toxins or pathogenic organisms, elevate a number of proinflammatory cytokines which have been associated with increased incidence of ASD in clinical studies and ASD-like behavior in animal models, as further described below. Moreover, recent preclinical and clinical data indicate that fetal neuroinflammation in response to external factors caused by maternal immune activation (MIA) and the resulting elevation of cytokine signaling may be important risk factors in the pathogenesis of ASD (reviewed in [4]).

Cytokines are low-molecular-weight glycoproteins that coordinate a variety of immune system responses. Cytokines serve a number of functions during innate and adaptive immunity, acting as key signaling molecules that regulate processes involved in induction and resolution of inflammation. Transmembrane cell surface receptors are responsible for the action of cytokines on cells. The binding of the cytokine to the receptor activates an intracellular signaling transduction pathway specific to the ligand:receptor complex, leading to the induction of transcription and the synthesis of new cellular proteins that can result in the differentiation of naïve immune cells and activation of various cell types within the microenvironment. The majority of cytokine receptors utilize one of the Janus kinase (JAK) family of molecules then activate the signal transducers and activators of transcription (STAT) family of proteins that regulate inflammatory signaling in a variety of somatic cell types. Throughout the human body, the proinflammatory cytokines interleukin-6 (IL-6) and interferon-γ (IFN-γ) are primarily secreted by activated immune cells as well as stromal cells and act on distinct STAT pathways (reviewed in [5,6]). In the brain, they are mainly secreted by specialized immune cells referred to as microglia, a type of monocyte found in the central nervous system (CNS) [7]. IL-6 can be secreted by both immune (macrophages, T-cells, B-cells, neutrophils, and microglia) and non-immune cells (muscle cells, adipocytes, fibroblasts, endothelial cells, and neurons) [8–10], whereas IFN-γ and many other cytokines are predominantly secreted by immune cells such as macrophages and antigen-presenting cells (APCs) [11].

Early reports suggested a strong association with high levels of proinflammatory cytokines, namely IL-6 and IFN-γ, in patients diagnosed with ASD [6,12–15], which have been implicated in the pathophysiology of these neurobehavioral diseases [16]. IFN-γ has been well described to potently induce transcriptional up-regulation of IL-6 in a number of cell types and contexts through IRF-1 and NF-κB [17–19]. Interestingly, while IL-6 acts as a negative regulator of IFN-γ in modulating T-cell activation [20], it has also been shown to be both necessary and sufficient to regulate IFN-γ-dependent immune responses to infection [21–24] and autoimmunity [25], suggesting a more complex relationship between these two cytokines that likely involve a number of cell types during the induction and resolution of inflammation. In line with this, IL-6 and IFN-γ have been shown to directly or indirectly activate the other’s canonical targets, STAT3 and STAT1, respectively, and regulate downstream transcriptional activity [25–30], indicating both coordinated and compensatory mechanisms to mediate inflammatory responses. Notably, STAT3 complex formation with JAK in response to IFN-γ stimulation has also been predicted through computational modeling to be STAT1 dependent, suggesting the possibility that IFN-γ activation may promote STAT3 recruitment and consequent IL-6-dependent transcriptional activity of STAT3 [28].

**Figure 1:**
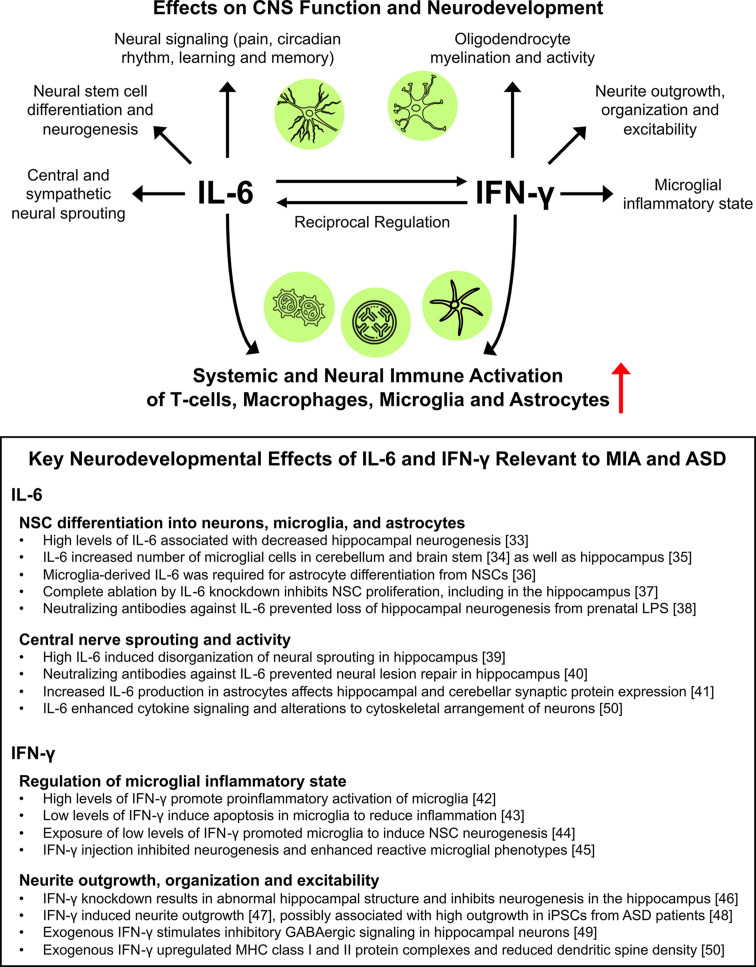
Overview of the effects of IL-6 and IFN-γ on neural function, development, and inflammation Top: The described unique effects of both IL-6 and IFN-γ on neural signaling and function, neural stem cell differentiation and neurodevelopment. Bottom: IL-6 and IFN-γ have been identified as key cytokines that promote the activation of a number of cell types both systemically as well as within the CNS, including T-cells as well as microglia, astroctyes, and other monocytes. Box: Key neurodevelopmental effects of IL-6 and IFN-γ relevant to MIA and ASD**.** Summary of key data that evaluate early neurodevelopmental processes that are altered using transgenic and pharmacologic approaches to modulate IL-6 and IFN-γ signaling. Brief explanations of major findings are included with specific literature references included in brackets.

Importantly, in the context of ASD, IL-6 and IFN-γ have been separately shown to regulate a number of neurodevelopmental processes, including neural stem cell (NSC) differentiation, outgrowth, sprouting, signaling and activity, and both serve as important mediators of neuroinflammation through regulating the activation of resident microglia, astrocytes, macrophages, and T-cells (reviewed in [31,32]). Use of transgenic mouse models to upregulate or ablate IL-6 and IFN-γ have provided evidence indicating that the balance of these cytokines is critical to preventing pathological abnormalities during early neurogenesis and gliogenesis in several regions that are associated with cognitive performance (e.g., learning and memory) and social behavior [33–50]. As summarized in Figure 1, data frequently indicated a biphasic relationship for both IL-6 and IFN-γ on neurodevelopment, with low levels often conferring protective effects favoring normal physiologic development while high levels have been associated with neuroinflammatory processes that may cause neural abnormalities, further supporting that tight control of these cytokine levels is critical in early development of the CNS. Taken together (Figure 1), it is possible that their interactions, whether direct or indirect, may potentiate neuroinflammatory-dependent developmental diseases. Critical to autoimmune-related neurobehavioral diseases such as ASD, both IL-6 and IFN-γ have been shown to be potent activators of inflammatory responses of microglia in the CNS [51,52], which in turn secrete high levels of both of these cytokines in addition to a number of others [31,51,53,54]. This strong proinflammatory microenvironment in the CNS, mediated in part by microglia as well as by IL-6 and IFN-γ signaling among other immune cell types, has been associated with altered cognitive and behavioral development, as described further. Given that (1) both IL-6 and IFN-γ have been reported in numerous cohorts to be highly expressed in ASD patients; (2) they both mediate neuroinflammation in CNS microglia and other immune cells; and (3) they have been shown to directly regulate expression and activity of the other, it is likely that the cross-talk of these cytokines may be of particular interest in ASD pathogenesis in response to inflammation during development. Herein, we review clinical and preclinical data supporting the potential interplay between IL-6 and IFN-γ, which may serve a considerable role in MIA-dependent ASD development.

## Immune activation and its potential role in ASD pathogenesis

Systemically, while macrophages and T-cells are a predominant source of IL-6, it is clear that multiple cell types produce IL-6 during the innate and adaptive immune responses [5,55]. In the CNS, activated microglia, astrocytes, and neurons have been described to markedly upregulate IL-6 production [31], in addition to other proinflammatory cytokines. While evidence support that immune activation may play a significant role in ASD, recent meta-analytic assessment suggested that an elevated Th17:Treg ratio occurs frequently in patients with ASD but found no significant differences in abundance of other cell types (cytotoxic CD8+ T-cells, B-cells and natural killer cells) [56]. While these data do suggest that proinflammatory T-cell signaling may be critical in the potential neuroinflammatory processes during development, it also indicates a complex relationship between immune activation and neurodevelopmental pathologies. In contrast with that meta-analysis, recent *ex vivo* evidence have demonstrated enhanced inflammatory signaling and reactivity of other immune cell types (e.g., B-cells, monocytes, neutrophils) derived from patients with ASD (discussed below). These data do support that immune cell activity in ASD is altered and the cross-talk among multiple cell types likely contributes to disease onset and progression; however, further evaluation is needed to fully understand the involvement of various immune cells in the etiology of ASD.

Regardless, T-cells that are phenotypically proinflammatory (e.g., Th1, Th2, and Th17) have been frequently observed to be altered in patients with ASD in multiple studies, and as noted above, the balance of T-cell phenotypes may be an important factor in disease progression [56,57]. T-cell responses to antigens require a complex formed by a T-cell receptor, or TCR, heterodimer and a CD3 dimer. This complex is responsible for recognizing and reacting with the major histocompatibility complex (MHC) cell surface glycoproteins expressed on APCs, as well as most somatic cells (MHC class I) and immune cells such as macrophages (MHC class II), whose subunits are encoded by the human leukocyte antigen (HLA) family of genes [58]. Once the TCR/CD3 complex recognizes the MHC, the TCR engages with the peptide fragments that are found on the MHC complexes. When looking at various models, the mechanosensory model showed that the TCR complex uses mechanical energy composed from T-cell engagement with the APCs to transmit signals [59]. The CD3 component of the TCR complex will then ultimately activate T-cells to cause differentiation and proliferation of subsets of effector T-cells. These in turn secrete cytokines and induce cell-mediated apoptosis of target cells to remove pathogenic or antigenic cells. The more the T-cells are exposed to an antigen, the lower the threshold for them to activate against that particular antigen in the future, which may be of particular interest in the context of autoimmune targeting of cytotoxic T lymphocytes [60]. Importantly, several cytokines secreted by activated T-cells are capable of passing the blood–brain barrier, generally through active transport [61,62]. A balance forms among these cytokines that counter-regulate each other, and it is suggested that any disruption to that balance can contribute to neurodevelopmental irregularity [63].

Changes to T-cell function can lead to immune irregularity, a prominent presence in patients diagnosed with ASD [63]. One of the primary effector cytokines is IL-6, which is secreted by activated T-cells in addition to a number of other immune cell types at the site of tissue damage or infection. While the major role of IL-6 is in proinflammatory signaling, it is also responsible for neural development and can cause the activation of other immune regulatory components [31]. IL-6 has an influential role in natural cell proliferation, migration, and synaptic connections. IL-6 levels tend to drop after an infection has been cleared from the body [64], and its expression/plasma levels are indicative of ongoing inflammation in tissues [5]. Notably, naïve and memory T-cells that differentiate into the Th17 phenotype, largely due to IL-6 signaling in conjunction with transforming growth factor-β (TGF-β) and interleukin-23 (IL-23), secrete high levels of IL-17 in addition to other cytokines ([65] and reviewed in [66,67]). Elevated IL-17 signaling in the CNS in turn activates astrocytes that provide a positive feedback loop through hypersecretion of IL-6 that, among other activities, further induce T-cell activation [68] as well as promote microglial proliferation and differentiation to proinflammatory M1 phenotypes [51]. IL-17 has also been shown to directly induce reactive microglial phenotypes which promotes their secretion of IL-6 (as well as IL-1β and TNFα), which further enhances Th17 differentiation and CNS inflammation [67]. Consistently, in addition to high levels of IL-6 and IFNγ observed in ASD patients described earlier, an abundance of Th17 cells have been frequently associated with ASD pathogenesis [56,69] as well as other neuroinflammatory autoimmune diseases [67]. Together, these clinical and preclinical data support the importance of IL-6 signaling, and its regulation by IL-17, as a contributing factor in developmental abnormalities through increased reactivity of astrocytes and microglia in the CNS sustained by T-cell activation.

**Figure 2:**
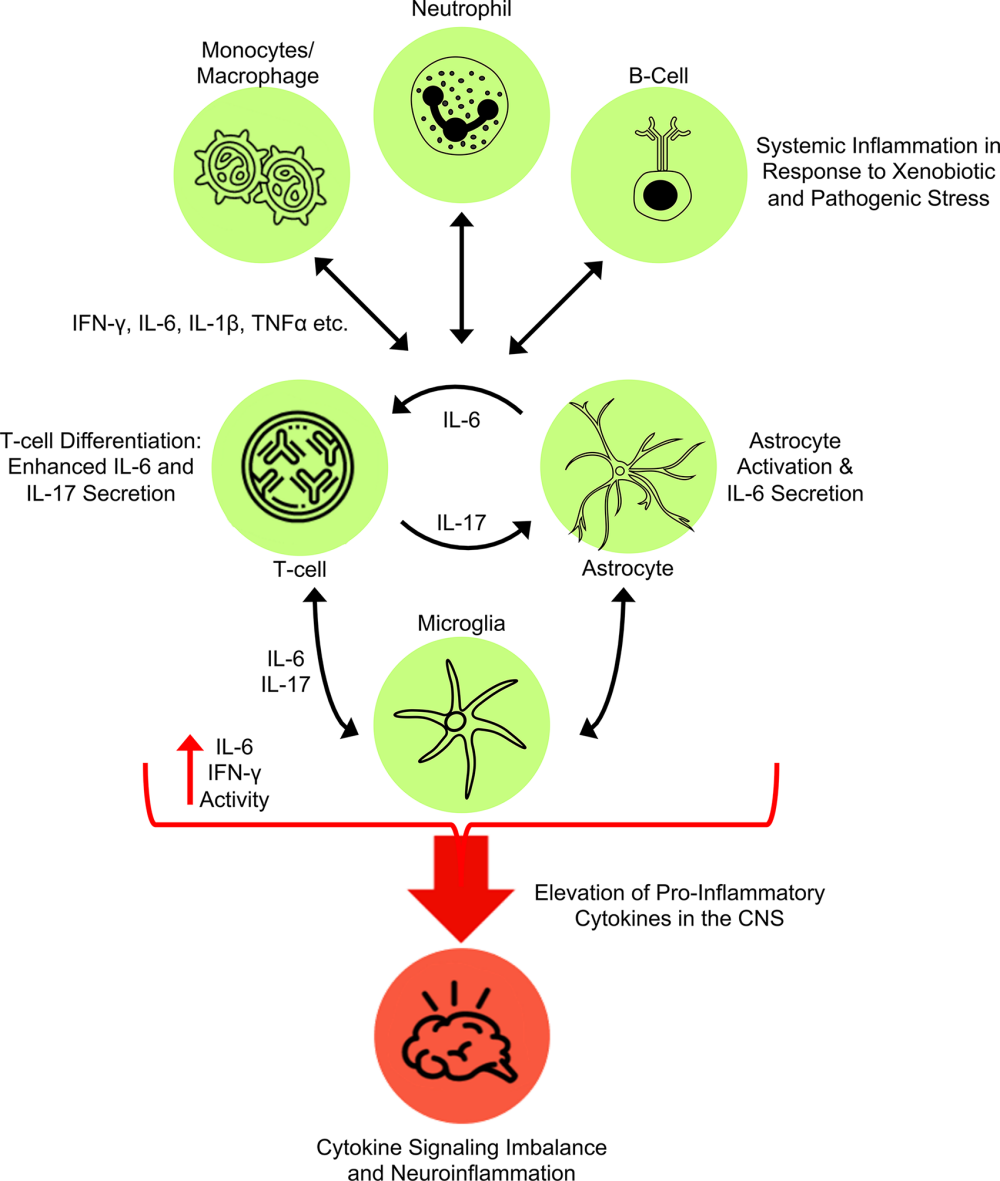
Immune cell cross-talk promotes neuroinflammation Numerous immune cell types (e.g., T-cells, B-cells, neutrophils, monocytes, macrophages) secrete cytokines that promote proliferation and differentiation in immune cells systemically and in the central nervous system (CNS). Systemic inflammation in response to xenobiotic and pathogenic stress can differentially activate the immune response that will alter both the systemic and central inflammatory milieu. The balance among cytokines will ultimately determine the number and phenotypic state of immune cells throughout the body and the CNS. In particular, T-cells activated by IL-6 and other cytokines secreted by immune cells differentiate to Th17 cells that are associated with ASD. These Th17 cells can promote activation of astrocytes, which in turn up-regulate IL-6 production forming a positive feedback loop between T-cells and astrocytes. Cytokines produced by these cells, as well as those of other systemic immune cells (e.g., monocytes, neutrophils, etc.) potentiate a proinflammatory environment characterized by high IL-6 and IFNγ (as well as IL-17 and other cytokines) that induce the activation of microglia that adopt a proinflammatory phenotype. This sustained high level of proinflammatory cytokines in the CNS causes an imbalance that can cause pathologic abnormalities during neurodevelopment.

Activated T-cells also produce an abundance of IFN-γ, which has diverse immune activity depending on the target cell type. Importantly, IFN-γ secreted by Th1 T lymphocytes has been shown to promote M1 proinflammatory polarization of macrophages, which in turn secrete several cytokines (e.g., IL-12) that induce the proliferation of Th1 T-cells and promote further production of IFN-γ (as well as IL-6) in immune cells [70,71]. IFN-γ also suppresses proliferation of Th2 T- cells responsible for feedback inhibition via IL-4 and IL-10, thus potentiating proinflammatory signaling [70]. Similar to IL-6, IFN-γ serves as a crucial diagnostic for assessing presence or severity of multiple diseases states [72] and, as expanded below, has been observed to have high plasma levels in patients with ASD. Taken together, immune cell activation and consequent communication between cytokines such as IL-6 and IFN-γ play a significant role in modulating systemic and central immune activity (Figure 2), and, given the evidence supporting elevated levels of these signals in both MIA and ASD, it is likely they are key to a central mechanism contributing to the etiology of ASD.

## Maternal immune activation (MIA) and autism spectrum disorders (ASD)

**Figure 3:**
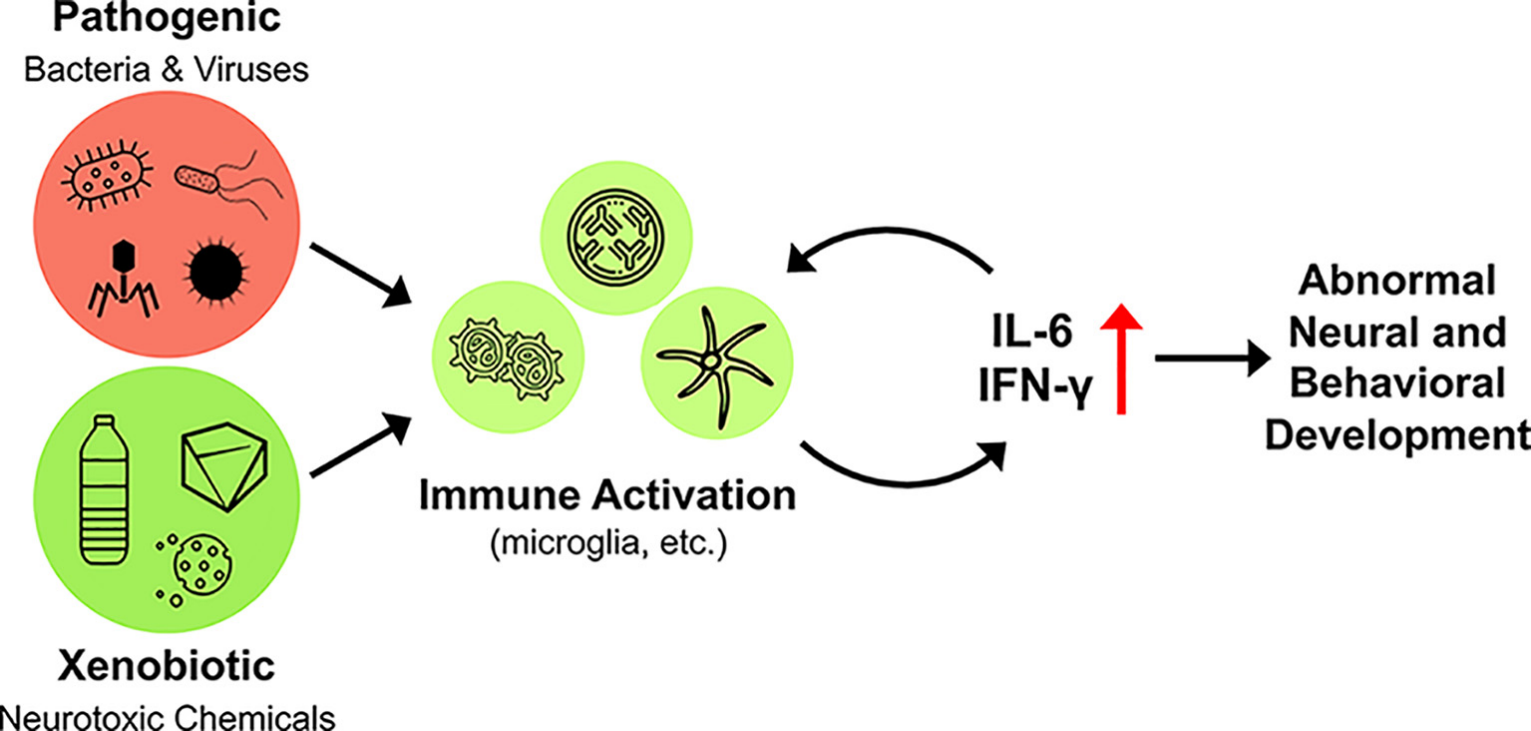
Environmental factors modulate IL-6 and IFN-γ and may impact their regulation of neurodevelopment Activation of immune cells (microglia, T-cells, and macrophages) by pathogenic and xenobiotic factors cause up-regulation of IL-6 and/or IFN-γ in *in vitro* and *in vivo* models. Pathogens in the studies provided are Zika virus, Dengue virus, and a number of bacteria, including *Listeria*, *Burkholderia Pseudomallei*, and *Brucella Abortus*. Xenobiotics in the studies provided include arsenic and DEHP [di-(2-ethylhexyl) phthalate]. Elevated IL-6 and IFN-γ have been directly associated with abnormal neurodevelopment and concomitant alterations in behavioral endpoints.

The possibility for proinflammatory immune activity to contribute to disrupted neurobehavioral function in progeny has been an area of rigorous research to understand the environmental and maternal contributions to ASD development. Maternal prenatal infections (bacterial and viral) and pyrexia, as well as other inflammatory disease states (e.g., autoimmune disease), have been implicated in a variety of developmental central nervous system (CNS) defects, including autism (recently reviewed in [73]). Infectious organisms including congenital rubella [74], and maternal prenatal measles, mumps [75], cytomegalovirus [76], bacteria [77], and influenza [75] have all been reported as risk factors in developing ASD. In the U.S.A., the prevalence of the first three viruses is minimal due to effective vaccination rates and therefore are unlikely to play a role in current cases of autism [78]. The effects of the influenza viruses on the developing fetus remain to be determined. In animal experiments, offspring of dams infected with human influenza viruses during pregnancy exhibited autistic-like behaviors suggesting the possibility of an association between influenza infection and risk of autism in humans [79]. Hospitalization due to influenza viruses are associated with an increased risk of development of ASD, especially in the first and second trimester [77]. Additionally, the maternal prenatal infection link has recently been reinforced by Zika virus (ZIKV) infections [80,81] with earlier association of neuropsychiatric disorders such as schizophrenia [82]. ZIKV, a mosquito-transmitted flavivirus, was identified as a cause of fetal brain injury when an outbreak in Brazil became associated with a surge in microcephaly [83]. Upon infection with ZIKV, a release of IL-6 and TNF-α ensues, which has detrimental effects on the fetal brain [84]. Maternal prenatal pyrexia, which induces a proinflammatory response leading to increased levels of cytokines, has also been implicated in the development of ASD [78]. The activated maternal immune response, transmitted to the fetus via maternal serum, placenta and amniotic fluid, is proposed to be the critical mediator between the maternal prenatal infection and altered fetal neurodevelopment. These clinical data regarding the ability of infectious diseases and consequent inflammation to promote neurodevelopmental pathogenesis are consistent with studies indicating an association of familial (maternal) autoimmune diseases and the incidence of ASD [85–88]. Taken together with data obtained from animal models (discussed further), it is likely that MIA is a central mechanism for neuropathological and behavioral changes in the offspring. As a number of infectious diseases and other environmental factors have been associated with significant systemic up-regulation of IL-6 and IFN-γ in patients [89–91] and in experimental models [24,52,92–101], it is likely that the interplay of these two cytokines may together modulate the influence of MIA on neurodevelopmental diseases such as ASD (Figure 3).

The mechanisms underlying the pathogenesis of ASD are still unknown, but many studies have identified different extrinsic factors that can potentially contribute to autism through MIA. However, MIA has been associated with an increase in cytokine synthesis and release, including IL-6 and IFN-γ among others (e.g., IL-1β, TNF-α) in response to infection and external factors during pregnancy both clinically and in experimental *in vivo* models of MIA [102–105]. Factors that lead to ASD have been proposed to be both genetic as well as environmental factors that the mother is exposed to during pregnancy that precipitate MIA. A study assessing exposure to diesel exhaust identified that this pathogen can be a factor associated with development of ASD as exposure to a certain level of diesel exhaust can disrupt the organization of the cortical lamina through dysregulation of extracellular matrix glycoproteins that regulates the process of neuronal migration [106]. Plasticizers like di-2-ethylhexyl phthalates (DEHP) are abundant in the environment and may also play a key role in pathogenesis of ASD. Chronic exposure to DEHP is associated with elevation of many proinflammatory cytokines such as IL-6 through upregulated NF-κB signaling. In a study by Nadeem and colleagues, it was observed that DEHP increased STAT3 (the primary downstream target of IL-6) mRNA and protein expression in monocytes of patients with ASD *ex vivo*, as well as up-regulated multiple inflammatory cytokines (IL-1β, IL-6, and TNF-α)[107]. These data suggest that DEHP has a high potential to exacerbate immune responses in patients with ASD through increased activity of proinflammatory transcription factors.

Exposure to metals such as inorganic arsenic, lead, aluminum, cadmium, and mercury has also been thought to influence neurodevelopmental disorders including the pathogenesis of ASD. Recent evidence suggest ASD-like social behavior in animals exposed to many of these neurotoxic metals *in utero* [108]. Interestingly, a recent systematic review and meta-analysis showed a significant association between metal exposure and ASD; however, these associations were not always positively correlated based on the levels of each of these metals between sampling sites (e.g., blood, hair and urine), and cadmium in particular was found to be negatively associated across all studies included [109]. This conflicting data may be in part due to the potential biphasic effects of such toxins in either activating or blunting the immune system, especially in the case of cadmium [110], or may indicate that other factors such as maternal metabolism or immune responsivity may influence the effects of these xenobiotics. Importantly, exposure to heavy metals such as arsenic, lead, cadmium and mercury have all been associated with increased immune activation, and specifically enhanced IL-6 and IFNγ production, in several preclinical and clinical contexts [111–116]; however, systematic *in vivo* studies evaluating these compounds’ effects on cytokine levels in the developing brain is still required. While there is a strong association between immune-activating external stressors (e.g., viral infections, pollution and other toxins) and neurodevelopmental abnormalities that may manifest as ASD, further preclinical and clinical research is needed to fully understand the mechanisms by which these extrinsic factors directly influence fetal neurogenesis and neurodevelopmental abnormalities.

**Figure 4:**
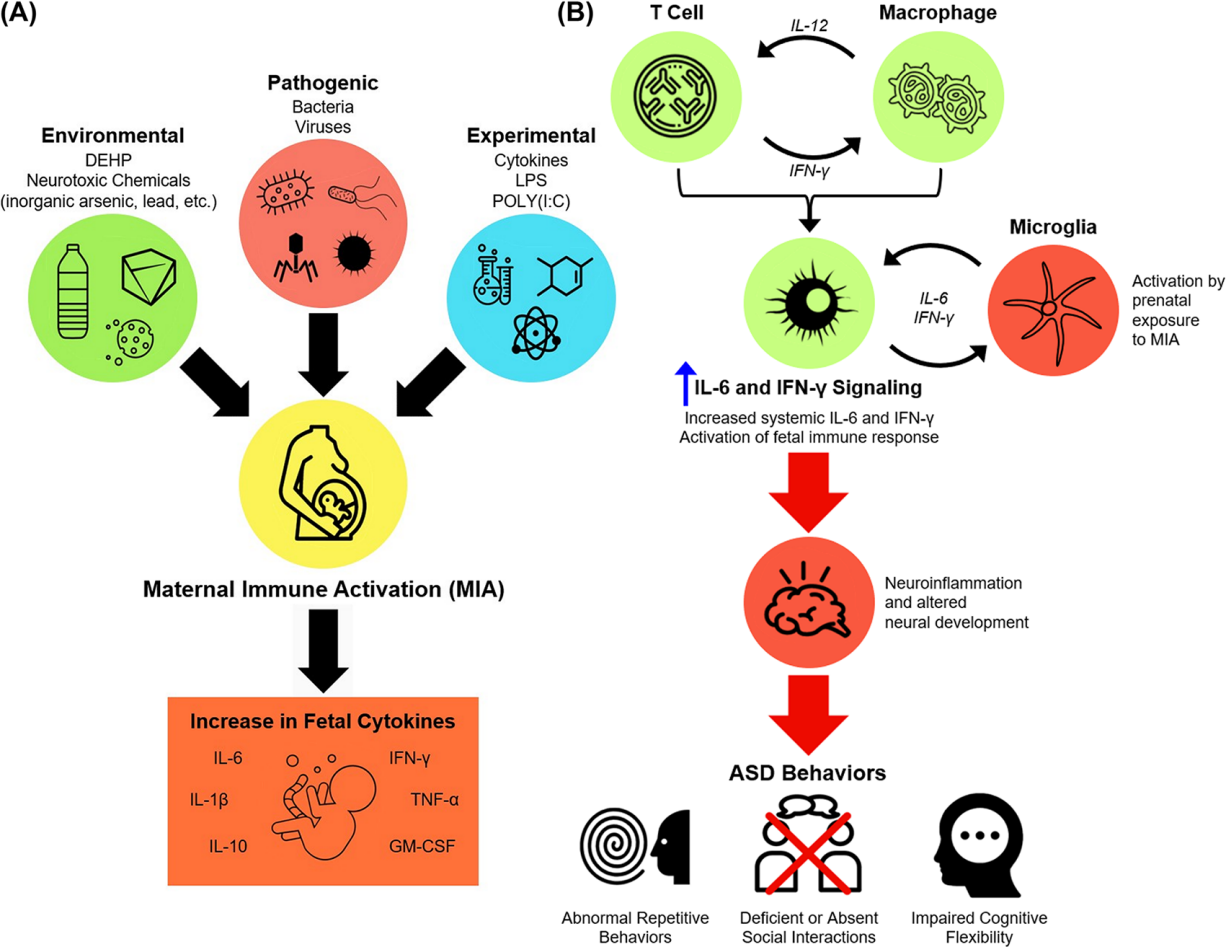
External factors may promote ASD through MIA-associated IL-6 and IFN**-γ** signaling **(A) Extrinsic factors that lead to maternal immune activation and increase levels of proinflammatory cytokines in offspring.** Extrinsic factors, including environmental, pathogenic, and experimental, can induce systemic inflammation during pregnancy referred to as maternal immune activation (MIA). As a result, systemic dissemination of elevated cytokines can cause subsequent immune responses in the fetus, and some (like IL-6) may potentially transfer through the placental barrier and accumulate directly. Abbreviations: ASD, autism spectrum disorder; DEHP, (di(2-ethylhexyl) phthalate; LPS, lipopolysaccharide; MIA, maternal immune activation; POLY(I:C), polyinosinic:polycytidylic acid. **(B) IL-6 and IFN-γ mediate maternal immune responses that promote gestational neuroinflammation and the development of autistic behavior in offspring.** Bidirectional signaling between T-cells and macrophages through cytokine signaling (e.g., IL-12 and IFN-γ) result in T-cell differentiation (via TCR signaling) and macrophage polarization to favor a proinflammatory state. Systemic (maternal) imbalance of proinflammatory cytokines such as IL-6 and IFN-γ can then activate microglia-mediated immune responses in the fetal brain. Neuroinflammation during development may result in ASD-associated behaviors, including abnormal repetitive behaviors, dysfunctional social communication/interactions, and impaired cognitive flexibility. Green circles indicate maternal/systemic immune activity; Orange circles denote fetal immune/neuroinflammatory activity.

In line with this, the MIA hypothesis (i.e., the contribution of inflammation during pregnancy to ASD) has been tested through a variety of methods including the immunostimulant polyinosinic:polycytidylic acid (poly(I:C)), being the most common, as well as bacterial lipopolysaccharide (LPS), direct injection of cytokines, and direct injection of microbial pathogens in *in vivo* models using mice and nonhuman primates (Figure 4A) [4,117–120]. The offspring of the subjects in these models are evaluated for changes in both brain development and behavioral changes, such as decreased social interactions, repetitive behaviors, and deficits in learning and memory, which are measured via standardized testing techniques [121]. This, coupled with the evidence of histologic changes in the CNS, allows for translation to humans.

Upon exposure to immunostimulants, cytokine concentrations increase in the fetal brain and maternal serum, placenta, and amniotic fluid [103,122–125]. The changes in cytokines are region specific and are hypothesized to be the reason for having long-lasting effects on neuronal function [103,120]. A prime example of this is the microglia, which have function in immunity, neurogenesis, apoptosis, and synaptic remodeling [126]. Microglia are activated by prenatal exposure to MIA, resulting in a localized release of cytokines, growth factors, and free radicals [127–131], leading to a long-lasting effect of increased levels of inflammatory mediators resulting in neurotoxicity [132]. To this effect, gestational MIA using poly(I:C) has recently been shown to markedly increase placental cytokines such as IL-6 and increase motility of microglia especially in response to additional immunostimulation using LPS [133]. This altered directionality of microglial movement was also strongly correlated with reduced social behavior in mice exposed to poly(I:C) *in utero* and subsequently challenged with LPS injection postnatally. These findings are especially important considering an early clinical study indicating elevated midgestational maternal cytokine levels (e.g., IFN-γ) in women who gave birth to offspring that were subsequently diagnosed with autism [134]. Therefore, induction of MIA resulting from prenatal infection and subsequent increases in proinflammatory cytokine expression may precipitate adverse neurological and behavioral outcomes in offspring that could manifest as ASD (summarized in Figure 4B) [135].

## Experimental assessment of IL-6 and IFN-γ in MIA-mediated ASD

Clinical research investigating how immune activation in the brains of patients diagnosed with ASD compared with patients who did not have the condition have presented intriguing information that indicate sustained neuroinflammation in patients with ASD. In patients diagnosed with ASD, it has been observed that there is an abnormal elevation of inflammatory markers within the mother while the patient was still in gestation [136]. It is also noted that these patients often exhibit altered T-cell function, particularly in their cytokine profiles, and that there is a higher number of monocytes in the plasma of ASD patients [136]. The cytokines, which are all proinflammatory, that were measured in these patients included IL-1β, IL-6, IFN-γ and TNF-α. Frozen brain tissue, specifically material located in the frontal cerebral cortex, in patients with ASD compared with controls revealed marked increases in several key proinflammatory cytokines (IL-1β, IL-6, IFN-γ, TNF-α and GM-CSF) and a concomitant suppression of the anti-inflammatory cytokine IL-10 [137]. Numerous *ex vivo* approaches have further evaluated the behavior of immune cells from patients to determine the role of inflammation in ASD development. Recently, it had been observed that B-cells isolated from patients with ASD exhibit increased production of IL-6 and decreased production of IL-10 (an anti-inflammatory cytokine) and were sensitized to challenge with LPS [138]. In a similar study, monocytes and PBMCs extracted from patients with ASD that were subsequently treated with LPS *ex vivo* also demonstrated enhancements to IL-6 production [139]. Enhanced inflammatory signaling was also shown in primary monocytes from patients with ASD marked by an up-regulation of IL-17, presumably downstream of IL-6 signaling, which was associated with enhanced oxidative stress and NFκB activation [140]. Similarly, neutrophils derived from ASD patients demonstrated increased levels of IL-6 and IL-17 expression as well as elevated NFκB activity, suggesting an overall enhancement in systemic inflammation in these patients [141]. Importantly, in T-cells obtained from patients with ASD it was observed that soluble IL-6 receptor was upregulated with concomitant increases to STAT3 activation and IL-17 production [142]. Together, these data are consistent with the idea that aberrant inflammation may be associated with the pathogenesis of ASD and that a number of cell types involved in innate and adaptive immunity may contribute to neurodevelopmental disorders.

Many *in vivo* approaches assessing fetal immune responses to inflammation have been conducted by injecting pregnant mice with the immunostimulants poly(I:C) or LPS. Especially in the case of poly(I:C), many studies have identified increased plasma levels and CNS expression of IL-6 and IFN-γ, as well as other cytokines (e.g., IL-17), in a number of *in vivo* approaches (for an exhaustive review, please see [143]). Mouse models of MIA have elevated proinflammatory cytokine levels during pregnancy which may impede normal brain development with the source of the excess levels having no effect on the behavioral outcome (whether it is endogenous or exogenous), supporting that MIA can result in a sufficiently elevated proinflammatory state that alters cytokine signaling within the developing fetal brain [144].

Another approach using BTBR T+ Itpr3tf (BTBR) mice, which mimic the behavior and other characteristics often present in an individual with ASD, has also been used to model inflammation-mediated behavioral abnormalities. The mutations inherent to these mice cause abnormal deformation of the corpus callosum and altered hippocampal functioning sometimes observed in patients with ASD [145]. Importantly, the mice display the three defining diagnostic symptoms of autism: abnormal repetitive behavior, deficient or absent social interactions and communication, and impaired cognitive flexibility [146], and have been successfully phenotyped through multiple *in vivo* assays assessing social behavior, memory, and stereotyped behavior [145]. Table 1 summarizes the experimental approaches used in the immunostimulant-dependent MIA models and the BTBR model of ASD with the corresponding data regarding immune response (i.e., IL-6 and IFN-γ expression) and behavior [103–105,122,146–163]. Some notable findings between these model systems are as follows:

- Poly(I:C) and LPS induction of IL-6 and IFN-γ in brain tissue of wild-type mice is associated with neural immune activation as well as abnormal repetitive behaviors and inhibited social behavior.
- Poly(I:C)-induced ASD-like behaviors requires IL-6 and IFN-γ (i.e., neutralizing antibodies inhibit immunostimulant-dependent abnormal repetitive behaviors).
- Poly(I:C)-induced abnormal repetitive behaviors could also be attenuated by blocking latent/delayed IL-17 activity, presumably upstream of IL-6.
- Recombinant IL-6 or IFN-γ are sufficient to promote abnormal repetitive behaviors in wild-type mice.BTBR mouse model of ASD demonstrates an overexpression of IL-6 and IFN-γ compared with wild-type mice, similar to that observed in wild-type mice treated with immunostimulants.
- Anti-inflammatory agents (AG126, VGX-1027, 5-AIQ, Resveratrol) in perinatal/juvenile BTBR mice reduce IL-6 and IFN-γ expression in immune cells and brain tissue, and are associated with reduced abnormal repetitive behaviors caused by these agents.
- BTBR abnormalities in social behavior is dependent on IL-6 and STAT3 activity and abnormal repetitive behaviors can be reversed in an IL-6-dependent manner.An immunomodulator that inhibits IFN-γ-dependent inflammation (VGX-1027) inhibits IL-6 in T-cells and brain tissue of BTBR mice.
- IL-6, STAT3 phosphorylation, and IL-17 up-regulation in splenic CD4+ T-cells and brain tissue is associated with abnormal repetitive behaviors in BTBR mice, which can be reversed through pharmacologic inhibition of Th17 immune responses.

**Table 1:** Overview of the effects of experimentally-induced MIA and experimental models of ASD on IL-6 and IFN-γ and social-behavioral indices

Model	Experimental approach	Alterations in IL-6 or IFN-γ	Behavioral changes	Ref.
MIA*	poly(I:C) i.p. in dams	Elevated IL-6 and IFN-γ in fetal and perinatal brain tissue	Poly(I:C) decreased pre-pulse inhibition and latent inhibition	[103]
	LPS i.p. in dams	Elevated IL-6 and IFN-γ in fetal brain tissue; increased microglial activation	LPS decreased exploration in social chamber [containing another animal]	[104]
	poly(I:C) i.p. in dams	Increased IL-6 expression and STAT3 phosphorylation in fetal brain tissue	IL6RA KO restored social exploration reductions from poly(I:C); IL6RA KO decreased marble-burying repetitive behavior caused by poly(I:C)	[105]
	poly(I:C) i.p. in dams ± anti-IL-17 neutralizing antibody	Increased serum IL-6 and IL-17 in response to poly(I:C)	Anti-IL-17 neutralizing antibody decreased marble-burying repetitive behavior downstream of IL-6	[122]
	recombinant IL-6 or IFN-γ i.p.; poly(I:C) i.p. in dams ± anti-IL-6 or IFN-γ neutralizing antibodies	Not measured	Recombinant IL-6 or IFN-γ decreased pre-pulse inhibition and latent inhibition; Neutralizing antibodies for IL-6 or IFN-γ restored pre-pulse inhibition deficits caused by poly(I:C)	[147]
	recombinant IL-6 i.p.; poly(I:C) i.p. in dams ± anti-IL-6 neutralizing antibodies	Not measured	poly(I:C) induced IL-6-dependent repression of sociability (by novel object/animal test)	[148]
**BTBR**	BTBR mice compared with C57 control mice (8–12 months)	Not measured	Increased movement in open field test and grooming; decreased sniffing and object exploration	[146]
	DEHP *ad libitum* in the water of newly weaned mice (3 weeks)	Increased systemic IL-6 and splenic STAT3 phosphorylation in BTBR mice but not control	Increased grooming and marble burying; decreased social exploration in BTBR mice but not control	[149]
	LPS ± IFN-γ on macrophages derived from control or BTBR mice (10–12 weeks)	Increased IL-6 in BTBR but not control macrophages	Decreased social exploration in response to LPS was potentiated by IFN-γ	[150]
	CGS21680 [agonist] or SCH5826 [antagonist] of ADORA2] i.p. (6–7 weeks)	Increased IL-6 and IFN-γ in brain tissue of BTBR mice compared with WT; mimicked by SCH5826 (81)	Not measured	[151]
	CGS21680 [agonist] or SCH5826 [antagonist] of ADORA2] i.p. (6–8 weeks)	Increased STAT3 phosphorylation and IL-17 production in BTBR mouse brain tissue and splenic CD4^+^ T-cells	ADORA2 agonism decreased grooming while antagonism increased grooming behavior	[162]
	AG126 TKI inhibitor i.p. in juvenile mice (7–9 weeks)	Increased IFN-γ in T-cells of BTBR mice compared with control; AG126 decreased IFN-γ in both mouse models (82)	Increased grooming and marble burying in BTBR mice restored to baseline (WT) levels by AG126	[152]
	VGX-1027 i.p. in juvenile BTBR or control C57 mice (6–7 weeks)	Increased IL-6 and IFN-γ in splenic CD4^+^ T-cells and brain tissue of BTBR mice; Decreased IL-6 and IFN-γ in CD4^+^ splenic cells and brain tissue of BTBR mice by VGX-1027, with no notable effects in C57 control mice	Increased grooming and marble burying in BTBR mice attenuated by VGX-1027; decreased social exploration restored to baseline WT levels by VGX-1027	[153]
	5-AIQ i.p. in BTBR or control C57 mice (undefined age)	Increased IFN-γ in splenic CD3^+^ T-cells and brain tissue of BTBR mice; Decreased IFN-γ in CD3^+^ splenic cells and brain tissue of BTBR mice by 5-AIQ, with no notable effects in C57 control mice	Increased grooming and marble burying in BTBR mice attenuated by 5-AIQ; decreased social exploration restored to baseline WT levels by 5-AIQ	[154]
	Resveratrol i.p. in BTBR or control C57 mice (6–8 weeks)	Increased expression of IL-6 and IFN-γ and STAT3 phosphorylation in splenic CD4^+^ T-cells and brain tissue of BTBR mice; Decreased expression of IL-6 and IFN-γ and STAT3 phosphorylation in CD4+ T-cells and brain tissue of BTBR mice by Resveratrol, with no notable effects in controls	Not measured	[155]
	sgp130Fc (IL-6 trans-signaling inhibitor) intraventricular injection (6–8 weeks)	Not measured	Increased sniffing time and decreased grooming in mice treated with sgp130Fc	[156]
	S3I-201 (STAT3 inhibitor) i.p. in BTBR or control C57 mice (6 weeks)	Increased IL-6 in splenic CD4^+^ T-cells and brain tissue in BTBR mice; attenuated in BTBR mice by S3I-201, with no notable effects in controls	Not measured	[158]
	S3I-201 (STAT3 inhibitor) i.p. in BTBR or control C57 mice (5-7 weeks)	Increased IFN-γ in splenic CD4^+^ T-cells in BTBR mice; attenuated in BTBR mice by S3I-201, with no notable effects in controls	Increased marble burying and grooming in BTBR mice; S3I-201 decreased marble-burying and excessive grooming in BTBR but not control mice	[159]
	Topical imiquimod in BTBR or control C57 mice (8–10 weeks)	Increased IL-6 and IL-17 in splenic CD4^+^ T-cells in BTBR mice; further increased by imiquimod-induced inflammation	Not measured	[160]
	DAPTA (CCR5 inhibitor) i.p. in BTBR or control C57 mice (7–8 weeks)	Increased IL-6 and IL-17 in splenic CD4^+^ T-cells in BTBR mice; attenuated in BTBR mice by DAPTA, with no notable effects in controls	Increased marble burying and grooming in BTBR mice; DAPTA decreased marble-burying and excessive grooming in BTBR but not control mice	[161]
	Sulforaphane (Nrf2 activator) i.p. in BTBR or control C57 mice (8–10 weeks)	Increased STAT3 phosphorylation and IL-17 expression in splenic CD4^+^ T-cells in BTBR mice, decreased by Sulforaphane	Increased marble-burying and grooming with decreased social exploration in BTBR mice, which was attenuated by Sulforaphane	[163]
**Other†**	sgp130Fc intracerebral injection in LPS-treated adult mice	sgp130Fc reduced STAT3 phosphorylation and IL-6 production of microglia and hippocampal cytokine (IL-6, IL-1β, TNFα) expression in LPS-treated mice	sgp130Fc restored social exploration of LPS-treated mice	[157]

Abbreviations: 5-AIQ, 5-aminoisoquinoline (PARP-1 inhibitor); ADORA2, adenosine A2a receptor; AG126, Tyrphostin AG126 ERK2 kinase inhibitor; BTBR, BTBR T+ Itpr3tf/J mouse model of ASD; CGS21680, agonist of ADORA2; DAPTA, D-ala-peptide T-amide CCR5 inhibitor; DEHP, di(2-ethylhexyl) phthalate; i.p., intraperitoneal; KO, knockout; LPS, lipopolysaccharide; IL6RA, IL-6 receptor alpha; poly(I:C), polyinosinic:polycytidylic acid; sgp130Fc, soluble gp130 decoy protein, an IL-6 trans-signaling inhibitor; S3I-201, STAT3 small molecule inhibitor; TKI, tyrosine kinase inhibitor; VGX-1027, immunomodulator that inhibits IFN-γ signaling. *For a comprehensive review of MIA mouse models/approaches for behavioral modeling of ASD and other neurodevelopmental disorders, please see C.M. Solek et al., Developmental Dynamics, 247:588-619, 2018. † ‘Other’ includes manuscripts assessing pathways involved in immunostimulant-induced behavioral abnormalities with specific relevance to IL-6 or IFN-γ signaling.

Overall, the current preclinical data supports the notion that IL-6 in particular is a central cytokine responsible for modulating immunological responses to environmental injury and orchestrate neuroinflammatory response to pathogenic and xenobiotic stress. While IFN-γ has also often been observed to be elevated in animals displaying ASD-like behavior and induces immune activation and abnormal social behavior in mice, further studies are required that specifically evaluate IFN-γ using more direct genetic and pharmacologic techniques (e.g., IFNR knockdown or neutralizing antibodies against IFN-γ, respectively) in order to determine its specific impact on IL-6 signaling and subsequent MIA-dependent ASD. It could prove vital to take a sophisticated approach in modulating these cytokines and their receptors in a cell-dependent manner both *in vivo* as well as in co-culture systems *in vitro*, which may further elaborate the complex relationship between immune cells and inflammatory signaling mediated by IFN-γ and IL-6 during neurodevelopment. Regardless, taken together the current data indicate that environmental immune activation (i.e., through poly(I:C)) or direct modulation of IL-6 and IFN-γ signaling strongly regulate immunologic profiles and *in vivo* social behavior relevant to ASD. While promising, investigations are required that utilize approaches that combine prenatal environmental (e.g., xenobiotic and pathogenic) stress in combination with manipulation of IL-6 and IFN-γ (as well as other suspected signaling molecules) in order to fully elucidate the signaling mechanisms underlying the contribution of MIA to ASD pathogenesis. Moreover, data evaluating the exact nature of these and other cytokines during normal physiologic fetal development remain sparse, and as a result further limit the translatability of current findings to the clinical context of ASD development.

## Potential interplay between IL-6 and IFN-γ signaling, MIA and ASD

Preclinical data indicate that dysfunctional IFN-γ signaling may lead to pathogenic immune cell function in response to antigens during development, as described above. In line with this, early studies using *in vivo* models indicate that mice lacking competent IFN-γ signaling develop hyperreactivity to multiple sources of xenobiotic injury [164–166]. Alterations in HLA signaling in response to IFN-γ could have significant consequences for immune activation given the importance of this pathway in regulating T-cell activity [58]. As the combination of HLA subunits within an MHC complex will determine the breadth of antigens (endogenous or exogenous) that can elicit a response as well as the degree of response triggered by any given antigen, the highly polymorphic nature of HLA genes are inherently linked to increasing the diversity of antigens that can be recognized and directly impacts immunogenic activity [58]. Indeed, the association between HLA genes and autoimmune disorders is not a new concept [167], and has been shown in multiple cases to contribute to genetic predisposition of developing some neurological autoimmune diseases [168]. Importantly, numerous recent studies have reported alterations in HLA variable regions in patients diagnosed with ASD, some of which occur at sites with mutations present in other autoimmune diseases [169–171]. While it is unclear whether the genetic variance observed in ASD in HLA signaling downstream of IFN-γ may directly relate to specific altered functional immunologic responses to inflammation (i.e., whether gain or loss of function in experimental conditions), this presents the possibility that variation in HLA regions may influence deleterious IFN-γ signaling in individuals. One consequence of this could be aberrant IL-6 signaling during MIA that influences neuroinflammation during development. Taken together with the ability of IFN-γ to regulate IL-6 expression and for these two cytokines to have somewhat redundant and reciprocal signaling [25,27], particularly in the context of pathogen-induced immune activation [71], it is possible that genetic perturbations observed clinically in HLA/IFN-γ may predispose individuals to immune reactions that alter the IL-6/STAT3 signaling intrinsically necessary for both neurodevelopment as well as response to inflammation during neurogenesis and neural signaling. The potential reciprocal regulation of IFN-γ by STAT3 activation, which may be important in ASD patients as observed in the BTBR mice [159], also indicates that the interactions between IFN-γ and IL-6 may be a key target for preventing MIA-induced ASD development.

Further studies are required to understand the impact of HLA variation observed in patients with ASD on molecular interactions between IFN-γ and IL-6 signaling between neurons and resident immune cells, especially in an *in vivo* context, and may help elucidate whether neuroinflammation resulting from xenobiotic stress may be a driver of the dysregulated CNS signaling associated with ASD behavior. Such studies may identify approaches for diagnostic testing to stratify patients based on risk of having severe MIA in response to external factors, and if MIA does in fact significantly contribute to the neurodevelopmental abnormalities that manifest as ASD, then this could provide a critical window of opportunity for therapeutic intervention to prevent ASD pathogenesis. A deeper understanding of how HLA polymorphisms may affect consequent cross-talk between IFN-γ and IL-6 signaling may provide novel insight into the interface of gene-environment interactions that may be critical to the degree of MIA and the potential development of ASD. However, as noted above, it is still essential for preclinical studies to fully delineate the basic relationship between IFN-γ and IL-6 during fetal exposure to environmental factors and how these signaling molecules may regulate MIA and alter neurodevelopmental processes.

## Concluding remarks

There are clear indications that the etiology of ASD is closely linked to both genetic and environmental factors that influence the degree and duration of inflammation resulting from MIA. While it is indeed probable that multiple cytokines and the evolving immune milieu during different points of fetal development play a role in neurodevelopmental dysfunction, current preclinical and clinical data strongly support a significant involvement of IL-6 and IFN-γ. Mechanistically, it is likely that the bidirectional signaling of these molecules between T-cells, microglia and other immune cell types are central to severe MIA that result in aberrant neuroinflammation that may cause ASD. Thus, the possibility for inhibiting the cross-talk between IL-6 and IFN-γ in preventing neurodevelopmental disorders that are potentially inflammation-dependent such as ASD presents an attractive therapeutic approach. In the case of the former, the IL-6 receptor antagonist Tocilizumab has had some clinical success in treating refractory autoimmune encephalitis [172], a disease that is strongly associated with gestational inflammation [173] and is observed in a majority of children with ASD [3]. However, the drug itself has demonstrated embryonic toxicity *in vivo* [174], decisively limiting its usefulness in the context of MIA. Therefore, the way forward in preventing occurrence of MIA-dependent ASD could be through repurposing anti-inflammatory drugs with low toxicity profiles that target either or both IL-6 and IFN-γ in patients at high risk of severe MIA. Preclinical and clinical evaluations of such approaches may elucidate novel therapeutic strategies to prevent MIA-dependent neuroinflammation and subsequent pathogenesis of ASD.

## Abbreviations

5-AIQ: 5-aminoisoquinoline
ADORA2: adenosine A2a Receptor
ASD: autism spectrum disorder
BDNF: brain-derived neurotrophic factor
CD3: cluster of differentiation 3
CD4: cluster of differentiation 4
CD8: cluster of differentiation 8
CNS: central nervous system
DEHP: di(2-ethylhexyl) phthalate
GM-CSF: granulocyte-macrophage colony-stimulating factor
HLA: human leukocyte antigen
IFN-γ: interferon-γ
IL-10: interleukin-10
IL-17: interleukin-17
IL-1β: interleukin-1β
IL-23: interleukin-23
IL-4: interleukin-4
IL-6: interleukin-6
IL-6RA: interleukin-6 receptor α
i.p.: intraperitoneal
IRF1: interferon regulatory factor 1
JAK: janus kinase
LPS: lipopolysaccharide
MHC: major histocompatibility complex
MIA: maternal immune activation
NF-κB: nuclear factor κB
Nrf2: nuclear factor erythroid 2-related factor 2
NSC: neural stem cell
PARP: poly(ADP-ribose) polymerase
poly(I:C): polyinosinic:polycytidylic acid
RORγT: RAR-related orphan receptor γ
SNP: single nucleotide polymorphism
STAT3: signal transducer and activator of transcription 3
TCR: T-cell receptor
TGF-β: transforming growth factor β
TKI: tyrosine kinase inhibitor
TNF-α: tumor necrosis factor α
